# Exploring strategies for enhancing access to oral healthcare for adults in Australia: a scoping review

**DOI:** 10.3389/froh.2025.1669597

**Published:** 2025-11-06

**Authors:** Chaminda Jayasekara Liyana Patabendige, Nicolie Jenkins, Christina Malatzky, Alexia Rohde, Kelly McGowan, Sundresan Naicker

**Affiliations:** 1 Australian Centre for Health Services Innovation and Centre for Healthcare Transformation, School of Public Health and Social Work, Faculty of Health, Queensland University of Technology, Brisbane, QLD, Australia; 2 School of Public Health and Social Work, Faculty of Health, Queensland University of Technology, Brisbane, QLD, Australia; 3 School of Public Health and Social Work, Centre for Justice and the Centre for Decent Work and Industry, Faculty of Health, Queensland University of Technology, Brisbane, QLD, Australia; 4 Critical Care Program, The George Institute for Global Health, University of New South Wales (UNSW), Sydney, NSW, Australia; 5 West Moreton Hospital and Health Service, Queensland Health, Ipswich, QLD, Australia

**Keywords:** oral healthcare, access, Australia, adults, interventions

## Abstract

Publicly funded adult oral healthcare services are mostly excluded in Australia's universal health coverage, despite oral disease being among the most common and costly health problems. Australia's vast land area and significant cultural diversity represent further challenges to ensuring equitable access to oral healthcare. A scoping review with the objective of synthesising and describing interventions aimed at improving access to oral healthcare for Australian adults was conducted, guided by the Preferred Reporting Items for Systematic Reviews and Meta-Analysis extension for Scoping Reviews process. Four online databases (Web of Science, EMBASE, PubMed and CINAHL) and grey literature (via Google Advanced) were searched and multistage systematic screening and data charting processes were undertaken following the JBI manual. Thirty eligible records were identified. Eligible studies included the following target populations: First Nations, rural and remote populations (*n* = 10), homeless people and people with mental illness (*n* = 8), elderly communities (*n* = 6), public service consumers (*n* = 5), pregnant women (*n* = 4) and people with chronic diseases (*n* = 3). Studies included the following health workforce: dental care providers (*n* = 20), students and trainees (*n* = 5) and non-dental health professionals (*n* = 5). Interventions described at workforce level included: multidisciplinary care (*n* = 12), financial approaches (*n* = 7), expanded scope of practice (*n* = 7), academic collaborations (*n* = 5), public care coordination (*n* = 4) and technological applications (*n* = 3). The majority (*n* = 21) indicated successful interventions. Most studies (*n* = 11) included fewer than 40 participants or were pilot interventions (*n* = 10). The interventions described may be scaled to other similar settings. To achieve universal health coverage, innovative models emphasising flexible workforce skills, task-sharing and multidisciplinary care are needed.

## 1 Background

Oral health (OH) is more than the absence of disease in the mouth; it encompasses a standard of oral functioning that enables comfortable participation in everyday activities (1). OH is determined by complex interactions between social, economic, environmental, political, behavioural, biological and cultural factors (2) and plays a pivotal role in preventing chronic diseases including heart disease, stroke and type 2 diabetes (3). However, despite being largely preventable, tooth decay remains a prevalent global health issue (4). Factors such as high sugar and alcohol consumption, inadequate hygiene practices, infrequent dental check-ups, limited access to fluoridated water and dental services, prolonged wait times and high out-of-pocket costs (5, 6) all contribute to compromised OH.

Oral diseases, affecting 45% of the global population, are the most prevalent noncommunicable diseases worldwide with an estimated nearly 3.5 billion people impacted annually (7, 8). Poor OH has a profoundly negative impact on quality of life leading to stress, financial burden, anxiety, depression and diminished self-esteem (9). Improving access to oral healthcare services is thus important for overall health and wellbeing, which is reflected in current efforts globally to include OH in universal health coverage systems (10, 11).

In Australia, the publicly funded universal healthcare insurance scheme, Medicare, offers free or subsidised health services to its citizens and selected overseas visitors (12). However, dental services were largely excluded when Medicare was first established, which remains the case today (13). Unlike medical services covered under Medicare, publicly funded selected dental services are only accessible to specific groups within the community such as individuals holding concession cards (such as low-income individuals, senior citizens and people with disabilities) and children (up to 17 years) (12). Australia's dental services are administered by federal, state and territory governments, but predominantly via the private dental sector, resulting in variations in services (14). The Commonwealth currently supports Australian public dental services through different schemes. Each Australian state and territory operates its own public dental services, providing subsidised or free dental care for eligible individuals, with varying eligibility criteria across regions (14, 15). These services primarily serve low-income groups including pensioners and disadvantaged groups, offering essential dental care (14). However, limitations on and long wait times for these services often drive those who are eligible to seek private dental care (14).

With over 85% of oral healthcare services in Australia provided by private, for-profit dental clinics, including large corporations, private health insurance providers and individual dentists, cost disparities for consumers are significant (16). In 2020–21, the average Australian spent AUD$432 on dental services (17, 18). Private dental care often places a financial burden on individuals, prompting some (who can afford it) to invest in private insurance to manage financial risk (14). This system is associated with significant out-of-pocket expenses, particularly for advanced procedures, which may lead individuals to delay or skip necessary dental care (14, 19). The dental healthcare burden also strains the hospital system, with an estimated 83,000 preventable dental condition-related hospitalisations across Australia in 2020–21 (14, 15), mostly among children and First Nations Australians (20). Remoteness also plays a significant factor with rates of preventable dental hospitalisations seen to rise with increasing geographical remoteness (14). In Australia, oral healthcare challenges disproportionately impact rural and remote residents, who face additional challenges such as limited access to fluoridated drinking water and increased costs associated with healthy dietary choices and oral hygiene products (21). These circumstances are often compounded by other factors including lower income and socio-economic marginalisation (14, 21).

Australians experience substantial disparities in oral healthcare services due to the geographical vastness, skilled OH workforce and infrastructure are often concentrated within metropolitan areas and away from regional settings with higher disease burdens (22, 23). Workforce dissemination inequalities in regional and disadvantaged areas in Australia has the same effect. Australia's OH workforce is facing increasing staff shortages, driven by various factors including population growth and aging demographics. Policy changes such as the Stronger Rural Health Strategy for improving Australian health aims to supply a quality workforce distributed based on community needs, all of which require a workforce that aligns with evolving healthcare needs (24, 25). Regional and disadvantaged areas suffer from workforce shortages due to factors like geographic isolation, limited professional development and inadequate support (26, 27). However, there is limited understanding of provider level drivers behind these disparities, particularly in terms of individual and organisational factors (28).

The latest National Oral Health Plan (2015–2024), the second of its kind, recognises “access” as one of its six foundational areas for action. Furthermore, the National Oral Health Plan places a significant emphasis on reducing oral healthcare inequalities across the Australian population, which encapsulates one of its two primary goals (21). Despite evidence indicating that improving population access to oral healthcare is a key health priority, to date it remains unclear which strategies are most effective in improving oral healthcare access for Australian populations. Research exploring the characteristics and attributes of different interventions employed across diverse geographical and cultural contexts in Australia is vital for improving future practices. Context-specific approaches may guide more effective interventions, as has been demonstrated in other health areas (29). The objective of this review was to identify and map interventions that aim to improve oral healthcare access within Australian adult populations. In doing so, these interventions were categorised according to target population, health workforce, type and category of intervention as it relates to workforce changes and/or oral healthcare service delivery.

## 2 Methods

### 2.1 Overview

A scoping review was conducted using the Preferred Reporting Items for Systematic Reviews and Meta-Analysis extension for Scoping Reviews (PRISMA-ScR) (30) and Arksey and O'Malley's five-step methodological framework for conducting scoping reviews (31). Scoping review protocols established by the Joanna Briggs Institute (JBI) for were used for selecting inclusion criteria and guiding data extraction (32). By doing so, the review process maintained a high level of rigor and consistency.

### 2.2 Identifying relevant studies

Search strategies were designed and conducted after an initial exploration utilising PubMed Medical Subject Headings (MeSH) and keywords with an experienced information specialist. The database searches were structured around four search terms: “oral”, “health”, “access” and “Australia”. Piloting the search strategies refined the research question and determined the criteria for study inclusion/exclusion (see Table 1). Individually adapted searches to accommodate the research question, methodologies and subject areas were conducted across four online academic databases: Web of Science, EMBASE, PubMed and CINAHL for their sufficient coverage of scholarly literature. The search terms employed in PubMed are detailed in Table 1, with similar approaches followed in the other databases. Grey literature was identified through Google Advanced searches using the same keywords with the first 100 results reviewed. A pilot screening of the first 300 results of grey literature showed that relevant sources were concentrated within the initial pages of the search results, whereas results beyond the first 100 were often duplicative or not relevant.

**Table 1:** Search strategy.

Keywords	Search terms
Oral	(mouth disease[MeSH Terms] OR oral*[Title/Abstract] OR dental*[Title/Abstract] OR tooth*[Title/Abstract] OR mouth*[Title/Abstract] OR teeth[Title/Abstract] OR periodont*[Title/Abstract] OR gingivitis[Title/Abstract] OR caries*[Title/Abstract])
Health	(health care delivery[MeSH Terms] OR health*[Title/Abstract] OR service*[Title/Abstract] OR administration*[Title/Abstract] OR management[Title/Abstract] OR community[Title/Abstract] OR research[Title/Abstract])
Access	(health disparity[MeSH Terms] OR access*[Title/Abstract] OR equal*[Title/Abstract] OR inequal*[Title/Abstract] OR barrier*[Title/Abstract] OR equit*[Title/Abstract] OR inequit*[Title/Abstract])
Australia	(australia*[Title/Abstract] OR queensland*[Title/Abstract] OR “western australia"[Title/Abstract] OR “new south wales"[Title/Abstract] OR victoria*[Title/Abstract] OR tasmania*[Title/Abstract] OR “south australia"[Title/Abstract] OR “northern territory"[Title/Abstract] OR “australian capital territory"[Title/Abstract])

The search terms from each key word were combined using the Boolean operator “AND” to refine and narrow the search results.

### 2.3 Eligibility criteria

Studies published between January 1, 2000 and September 1, 2025 were eligible for inclusion if they reported on interventions aimed at enhancing oral healthcare access among adults in Australia. Studies published in languages other than English or studies prior to the year 2000 were excluded. A full list of the inclusion and exclusion criteria applied in this review is provided in Table 2.

**Table 2:** Inclusion and exclusion criteria.

Inclusion criteria	Exclusion criteria
Adult population (18 years and over) in any state or territory in Australia	Studies describing interventions aimed at enhancing healthcare access in general (rather than those specific to oral healthcare)
Interventions aimed at improving access to oral healthcare services	Interventions implemented in inpatient healthcare settings for improving OH
Literature reporting short term and/or long-term outcomes of the health system interventions	Studies focused solely on OH promotion and oral disease prevention educative activities that don't address access to oral healthcare services
Literature reporting original research with any study designs and reports	Studies conducted with sole intention of improving oral healthcare access among children (up to 18 years of age)
	Studies describing implementation of population level or policy level interventions (e.g., water fluoridation)
	Reviews, commentary and opinion pieces

OH, oral health.

The following operational definitions guided the screening process to ensure alignment with the review's objective.

#### 2.3.1 Oral healthcare access

The ability of individuals to readily obtain necessary dental services, including check-ups, preventive care and treatment, without facing significant barriers such as financial constraints, geographical boundaries or lack of available service providers. This covers the affordability, acceptability, appropriateness, availability and approachability dimensions to healthcare “access”, as well as insurance coverage and socio-cultural factors that may impact individuals' ability to seek and receive oral healthcare (21, 33).

#### 2.3.2 Intervention

Purposeful initiatives or strategies aimed at improving oral healthcare access across various service levels including individual, institutional or community with the goal of helping to achieve health equity within the adult population (34). These initiatives may encompass a range of activities undertaken by diverse entities, including governmental bodies, non-profit organisations, healthcare providers and community groups. The primary objective of these interventions is to overcome context-specific barriers and improve accessibility to appropriate oral healthcare services, including preventive care and treatment and outreach services, thereby improving OH outcomes for individuals and communities. In this definition, national, policy or population wide interventions such as water fluoridation were excluded. While such interventions may improve overall OH outcomes, they do not directly address access to oral healthcare, which is the specific focus of this review. Interventions without active engagement by individuals or groups of healthcare workers, such as dietary advice and preventive advice given by non-health professionals were also not considered. However, studies that included preventive or educational components were included if they were part of a broader intervention (such as tele dentistry consultations or outreach programs) that actively addressed access to oral healthcare.

### 2.4 Study selection

To recognise literature relevant for addressing this study's research question and meeting the inclusion criteria, a multistage screening process was employed, encompassing title and abstract screening and thorough reviewing of full texts. The final search results from the four databases were exported to EndNote and duplicates were removed. Afterward, the remaining publications were exported to Rayyan (35), a web tool for screening studies, where any remaining duplicates were eliminated. EndNote was used for reference management and duplicates were removed using either EndNote or Rayyan. Rayyan was used for blinding the screening process.

A randomly selected subset of 35 articles was independently screened by four authors (CJ, CM, NJ and AR). Thereafter, the remaining articles were independently screened by at least two authors. Any further discrepancies were resolved through group discussions with four authors (CJ, SN, CM and NJ) until a consensus was reached. Full text screening was conducted by the lead author and a final decision regarding eligibility was reached through discussions. The same multistage review process was applied to the grey literature.

### 2.5 Charting the data

Data from eligible studies were integrated into a data extraction table. The fields for data extraction were adapted from the JBI template found in the JBI Manual for Evidence Synthesis (32). Recorded variables included: author name; study region; year of publication; study design and method (quantitative, qualitative or mixed); name and/or types of interventions; target adult population group; and context of implementation to identify, categorise and describe health system interventions and workforce associated factors. The reviewers conducted pilot testing and refinement of the data charting domains. Data charting was conducted using an Excel spreadsheet. An initial subset of studies (*n* = 6) were independently trialled using the data charting form by three researchers who met to develop consensus around the variables and validate the extraction protocol. Remaining data extraction was completed by the lead author and any disagreements were discussed in team meetings until consensus was reached.

### 2.6 Collating, summarising and reporting the results

The results were summarised as follows: (a) Characteristics of the literature, including publication year, study design and study setting; (b) Target populations for access to oral healthcare; (c) Targeted health workforce where appropriate; (d) Interventions described at workforce level; (e) Description of study findings that was reported in the included studies.

## 3 Results

**Figure 1:**
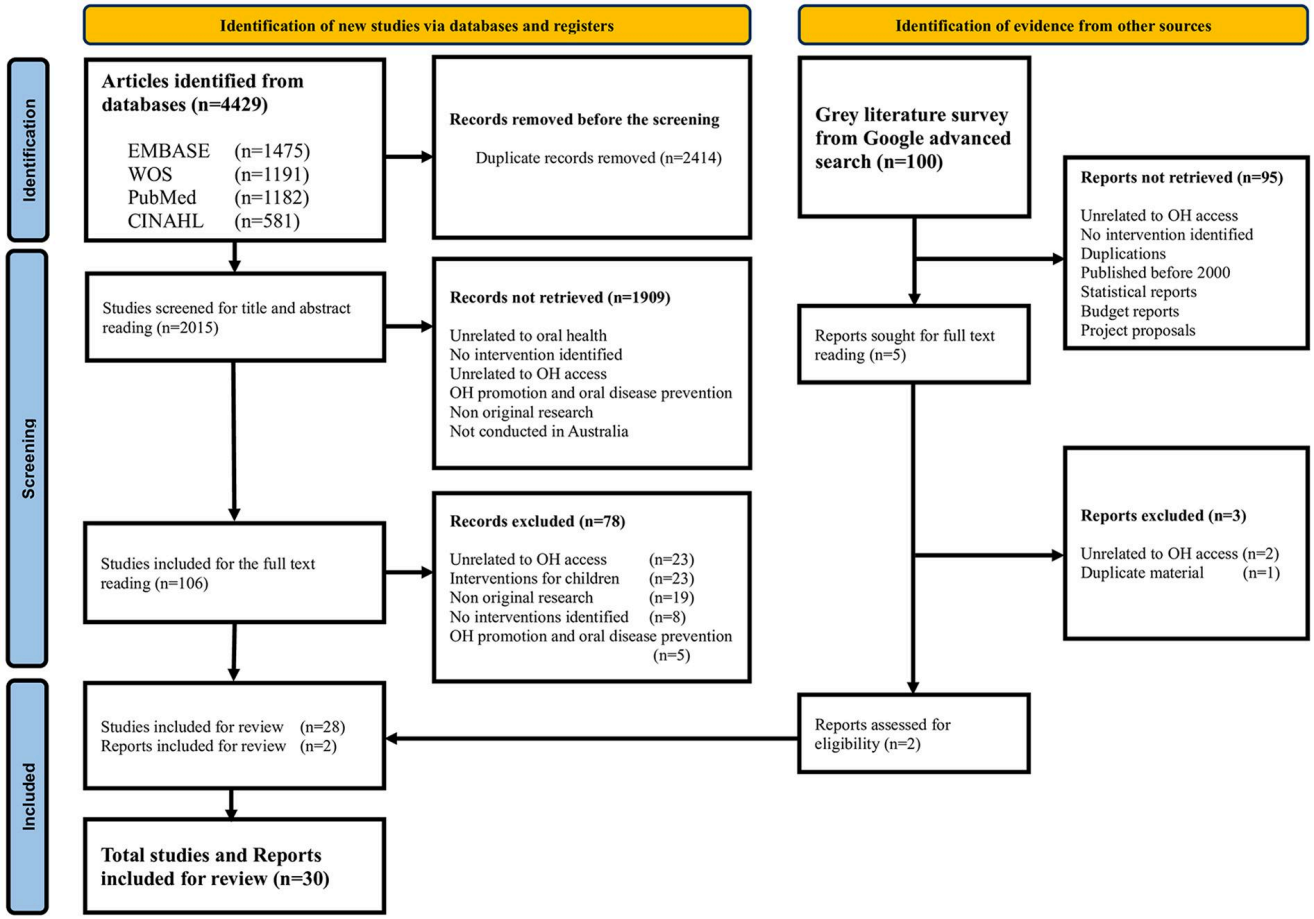
Preferred reporting items for systematic reviews and meta-analysis extension for scoping reviews.

The final academic database searches yielded 4,429 records (September 1, 2025). After removing duplicates (2,414 articles), 2015 studies underwent title and abstract screening. Subsequently, 106 articles were subjected to full text review of which 28 articles met all eligibility criteria. Additionally, a search of grey literature identified 100 reports. Of these, five were reviewed in full, while 95 excluded after reviewing web domains and titles. Two grey literature reports (36, 37) were included in this scoping review after full text review and provided insights on service delivery models and workforce-related initiatives not captured in the peer-reviewed studies. In total, 30 studies and reports met the inclusion criteria (see Figure 1).

### 3.1 Study characteristics

Identified studies reported interventions in diverse locations across Australia. An analysis of the geographical distribution of studies (see Table 3) showed a concentration in Queensland (38–46) with nine studies and Victoria (47–56) with ten studies. There were five studies (17%) related to Australia wide interventions (36, 37, 57–59). There were four studies (13%) based in New South Wales (47, 60–62) and two studies (6%) in Western Australia (63, 64). This review identified one study conducted in South Australia (65).The review revealed an increase in the number of access to oral healthcare related publications over time, with only one study (40) published between 2000 and 2009 and the remaining 29 publications (97%) from 2010 onwards (see Table 3). The peak publication activities occurred in 2018 (38, 41, 42, 52) and 2022 (37, 45, 53, 63) with four studies (13%) in each year. Of included studies, twelve (40%) utilised quantitative methods (39, 41, 43, 45, 47, 51, 53, 56, 58, 59, 61, 62), while nine (30%) employed qualitative approaches (44, 49, 52, 54, 55, 57, 63–65). Nine studies (30%) adopted the mixed methods approach (36–38, 40, 42, 46, 48, 50, 60) (see Table 3).

**Table 3:** Study characteristics and target populations (*n* = 26).

Characteristic	*n* (%)
Year of publications	
2000–2009 (40)	1 (3.3%)
2010–2019 (38, 41, 42, 47–52, 57, 58, 61, 62, 64)	14 (46.7%)
After 2020 (36, 37, 39, 43–46, 53–56, 59, 60, 63, 65)	15 (50%)
Methodological classification of studies	
Quantitative (39, 41, 43, 45, 47, 51, 53, 56, 58, 59, 61, 62)	12 (40%)
Qualitative (44, 49, 52, 54, 55, 57, 63–65)	9 (30%)
Mixed (36–38, 40, 42, 46, 48, 50, 60)	9 (30%)
Study setting^a^	
Australia wide (36, 37, 57–59)	5 (16.7%)
Victoria (47–56)	10 (33.3%)
Queensland (38–46)	9 (30%)
New South Wales (47, 60–62)	4 (13.3%)
Western Australia (63, 64)	2 (6.7%)
South Australia (65)	1 (3.3%)
Target populations^a^	
First Nations communities (36, 37, 44, 49, 50, 65) in rural and remote locations (37, 43, 44, 55, 59, 64)	10 (33.3%)
Homeless (36, 38, 39, 49, 50, 53, 63) and people with mental health conditions (49, 50, 52)	8 (26.7%)
RACF residents and elderly communities (40–42, 46, 61, 62)	6 (20%)
Public oral health service consumers (45, 51, 53, 54, 56)	5 (16.7%)
Pregnant women (47–49, 60)	4 (13.3%)
People with chronic diseases (57, 58, 65)	3 (10%)

RACFs, residential aged care facilities.

a The percentage exceeds 100 (*n* > 26) because the studies represent multiple domains.

Among the included studies, ten (33%) were pilot studies (38–40, 46–48, 51, 53, 60, 65) and nine studies (30%) used pre-post assessments (40–42, 45, 47, 48, 51, 60, 65). Seven studies (23%) included 20–40 participants (44, 46, 48, 54, 57, 63, 65), while four studies (13%) involved fewer than 20 participants (51, 52, 55, 60). Additionally, ten studies (33%) did not report participant numbers in relation to the respective interventions (36, 37, 43, 45, 49, 53, 58, 59, 61, 64) (see Supplementary Information).

### 3.2 Target populations

Included studies described a range of different intervention approaches aimed at improving access to oral healthcare, targeting groups with distinct needs (see Table 3). The largest proportion of studies (*n* = 10; 33%) focused on interventions for First Nations communities (36, 37, 43, 44, 49, 50, 65) in rural and remote locations (37, 43, 44, 55, 59, 64). Eight studies (*n* = 8; 27%) focused on interventions for homeless individuals (36, 38, 39, 49, 50, 53, 63) and people with mental health conditions (49, 50, 52). One fifth of studies (*n* = 6; 20%) represented different interventions focused on residents in aged care facilities (RACF) and elderly communities (40–42, 46, 61, 62). Studies describing interventions focused on public OH service consumers (*n* = 5; 17%) (45, 51, 53, 54, 56) and pregnant women (*n* = 4; 13%) (47–49, 60) were also identified in this review. Studies describing interventions targeting people with chronic diseases (57, 58, 65) represented the smallest proportion (*n* = 3; 10%) among all target groups.

### 3.3 Health workforces

The categories of OH workforce described in included studies were dental care providers (*n* = 20; 67%), students and trainees (*n* = 5; 17%) and non-dental professionals (*n* = 5; 17%) representing midwives, community pharmacists, Aboriginal health workers and nurses (see Table 4). The most frequent health providers engaged in included studies were dentists (*n* = 15; 50%) (36–39, 46, 49, 50, 54, 56–59, 61, 63, 64) and oral health therapists/dental therapists (*n* = 13; 43%) (37–42, 49–51, 54, 59, 62) followed by dental hygienists (37, 49, 65) (*n* = 3; 10%), dental prosthetists (37, 49) (*n* = 2; 7%) and dental assistants (37, 49, 54, 56) (*n* = 4; 13%). Dental specialists (49) were engaged in an intervention in only one study. The majority of trainees described in included studies were dental students (38, 39, 43, 44) (*n* = 4; 13%), working under supervision through public dental services. Two studies described the role of midwives (47, 48) in providing OH services for pregnant women in addition to their regular duties. An intervention engaging Aboriginal health workers to enhance access for culturally sensitive target populations was discussed in one study (60). Additionally, one study reported on community pharmacists in Victoria who provided oral health advice and consultations in collaboration with dental practitioners. These activities were supported through targeted training and promotional resources (55).

**Table 4:** Studies describing health workforce and interventions at workforce level (*n* = 26).

Health workforce	*n* (%)
Dental care providers	20 (66.7%)
Dental specialists (49)	
Dentists (36–39, 46, 49, 50, 54, 56–59, 61, 63, 64)	
Oral health therapists/dental therapist (37–42, 49–51, 54, 56, 59, 62)	
Dental hygienists (37, 49, 65)	
Dental prosthetists (37, 49)	
Dental assistants (37, 49, 54, 56)	
Students and trainees	5 (16.7%)
Dental students (38, 39, 43, 44)	
Dental nursing assistant students (63)	
Non dental health professionals	5 (16.7%)
Midwives (47, 48)	
Community pharmacists (55)	
Aboriginal health workers (60)	
Nurses (64)	
Interventions described at the workforce level^a^	*n* (%)
Multidisciplinary care (36, 37, 40, 48–50, 52, 54, 56, 59, 60, 64, 65)	12 (40%)
Financial approaches (36–38, 57, 58, 61, 63)	7 (23.3%)
Expanded scope of practices (37, 41, 42, 47, 51, 55, 62)	7 (23.3%)
Academic collaborations (36, 37, 39, 43, 44)	5 (16.7%)
Public care coordination (50, 57, 58, 61)	4 (13.3%)
Technology enabled care (45, 46, 53)	3 (10%)

a The percentage exceed 100 (*n* > 26) because the studies represent multiple domains.

### 3.4 Interventions described at the workforce level

Interventions at the workforce level revealed several key workforce strategies employed across different settings (see Table 4). There were 12 studies (40%) that employed multidisciplinary care as a strategy to improve access to oral healthcare. Multidisciplinary care is operationally defined as an integrated approach where healthcare professionals from diverse disciplines collaborate to provide patient centred care (66). Studies incorporated the following activities: a quality improvement initiative (40); care coordination with minimal intervention dentistry (49, 54) and informing respectful, empathic and culturally safe oral health practices with appropriate referrals for further care (48, 50, 52, 59, 60, 65). Additionally, new models of care through vertical integration of service and research to sustain services in remote locations (64), community service grants through partnerships to fund community-based OH projects and support volunteer dentists and dental students in high-risk areas (36) and early career support for rural graduate transition into career roles were also described (37).

Seven studies (23%) explored financial approaches as a strategy to improve access to oral healthcare, focusing on both direct and indirect financial assistance for patients. These financial strategies aimed to reduce economic barriers to care, facilitating greater access to essential OH services. Among these, strategies included pro bono interventions for better access to oral healthcare, where services are provided without charge (36, 37, 57, 61) and three studies highlighted financial assistance programs for care seekers from the private sector for free services to eligible adults under different schemes (57, 58, 61).

Seven studies (23%) described an expanded scope of practice as a workforce strategy aimed at improving access to OH services. Expanded scope of practice refers to the broadening of roles and responsibilities of healthcare professionals to include additional clinical tasks and services beyond their traditional scope for enhancing service delivery (67). Specifically, expanding the scope of practice among oral health therapists (41, 42, 62), university-educated dental therapists for independently treating patients aged 26 years and over (51) and community pharmacists was highlighted as a workforce strategy for addressing unmet need in underserved communities (55). Additionally, the involvement of a number of clinical schools (37) in facilitating this expanded scope of practice was recognised as a means to increase access to care for adults.

Academic collaborations were highlighted in 17% of the included studies (*n* = 5) as a key workforce strategy. Academic collaborations refer to partnerships between universities and health service providers aimed at enhancing healthcare delivery through education, training and service provision (68). Such collaborations were particularly evident in efforts to provide OH care to underserved populations, such as First Nations and homeless communities. Universities supported students in dental fields to enable them to serve their own communities (36, 37), through student led clinics and primary care outplacement programs in regional areas (39, 43, 44). These student staffed clinics were described as a practical solution to increasing care access, providing underserved populations with essential dental services while also training the next generation of OH professionals.

Four studies (13%) described workforce strategies related to public care coordination that included efforts to provide accessible care such as state fundings models, public dental services and federal subsidies for individuals with chronic diseases, as an approach for providing OH care to priority populations. These studies highlighted the public care coordination as a workforce strategy in providing the oral healthcare for priority populations (50, 57, 58, 61). Strategies included the implementation of a state funded model (61), a public dental service that emphasised building trust and delivering inclusive oral healthcare (50) and a federal government subsidy program for private dental treatment to support individuals with chronic illnesses (57, 58).

Three included studies (10%) described technological applications as workforce strategies aimed at improving access to oral healthcare (45, 46, 53). Technological applications refer to the use of digital tools and platforms to enhance the efficiency and reach of healthcare services. One study employed virtual dentistry to deliver remote oral healthcare services, including referrals for indicated public patients during the COVID-19 pandemic (53). Another study implemented real time tele dentistry (46), while the other study utilised short message service (SMS) reminders to reduce administrative burden of appointment scheduling and to improve consumer attendance (45).

### 3.5 Description of study findings

The interventions in the included studies were described based on their reported outcomes for improving access oral healthcare. Twenty five studies (38–48, 50–53, 55–63, 65) (83%) reported results describing intervention outcomes. Among them, there were 21 studies (38–44, 46–48, 50–53, 55, 56, 59, 60, 62, 63, 65) (70%) in which interventions were reported as improving access to oral healthcare. Of the studies observing positive effects, the majority employed quantitative methods (39, 41, 43, 51, 53, 56, 59, 60, 62) (*n* = 9; 30%), followed by mixed methods (38, 40, 42, 46, 48, 50, 60) (*n* = 7; 23%) and qualitative methods (44, 52, 55, 63, 65) (*n* = 5; 17%). Cost effectiveness was reported in four studies (13%) (39, 43, 58, 59) (see Supplementary Information).

The following describes study interventions that reported positive outcomes in relation to improving access to oral healthcare. Four studies described collaborative interventions between universities and public health services through dental student engagement (38, 39, 43, 44). Of these, three studies reported on patient initiated tele dentistry models that enabled remote oral healthcare services, including referral pathways for public service consumers (53) and services delivered by oral health therapists using tele dentistry platforms (41, 42). Other reported interventions included the introduction of evidence-based oral hygiene practices for elderly patients with dementia (40), the establishment of clinics across two clinical sites that provided preventive and restorative dental care for priority populations (50), volunteer-led, community-based oral healthcare programs that made services accessible for adults experiencing homelessness (63) and the use of a Health Prompt tool to initiate conversations about OH and support engagement with dental care among individuals living with mental illness (52). Three studies focused on models of care for pregnant women: a culturally safe program called the Grinnin' Up Mums & Bubs model of care (60) and the Midwifery Initiated Oral Health education program, an online evidence-based program developed for midwives (47, 48). One study described a pilot educational bridging program that enabled university educated dental therapists to expand their clinical scope (51), while another outlined the program, Reach-OHT program which provided structured oral healthcare to residents in RACFs through oral health therapists (62). Additionally, one study documented a fly-in, fly-out mobile and outreach service delivery model funded by the Commonwealth of Australia that expanded service reach in rural and remote areas (59).

Conversely, four studies (13%) described interventions that did not lead to reported improvements in accessing oral healthcare (45, 57, 58, 61). Three utilised quantitative methods (45, 58, 61) while the remaining study utilised the qualitative methodology (57). Three studies discussed funding mechanisms, implemented at the state level for the elderly population (65 years and above) (61) and nationwide for individuals with chronic disease conditions (57, 58), that did not demonstrate overall success. The remaining study explored the use of SMS reminders for public service consumers to reduce administrative burden and increase consumer attendance. However, this intervention was found to be unsuccessful (45). Interestingly, around one sixth of the studies (*n* = 5; 17%) did not report results related to intervention outcome (36, 37, 49, 54, 64).

## 4 Discussion

This study identified a wide range of interventions aimed at improving access to oral healthcare among Australian adults. A majority of the identified studies (97%) were published after 2010, indicating a growing interest for initiatives to improve oral healthcare access among the adult Australian population (36–39, 41–65). Conversely, the absence of studies from the Australian Capital Territory, Northern Territory and Tasmania, highlights potential disparities within the published literature. Furthermore, there were no randomised control trials or comparison arms in any of the included studies suggesting a significant gap in the quality of evidence currently present in this review (69). As a result, the wide and heterogeneous body of evidence collected made it challenging to synthesise findings across studies and grey literature, necessitating a descriptive scoping review rather than an evaluative systematic review.

Findings highlight the failure of public funding for private services. This was most notable for the Chronic Disease Dental Scheme (CDDS) which was ultimately deemed cost ineffective due to inappropriate use of funds which were not adequately gate-kept (57, 58). Internationally, similar issues have been observed, with these hybrid payment systems for adult oral healthcare (70). It appears unregulated private sector collaborations that lack gatekeeping or auditing do not work. Similarly, without fee regulation, there are concerns about “Too Much Dentistry,” (71) which can lead to overdiagnosis and overtreatment instead of appropriate evidence-based care. However it is important to highlight that approximately 85% of the oral healthcare services in Australia are provided by the private sector (72). This raises an important concern: it may not be possible to implement large scale OH interventions successfully in Australia without engaging the private sector.

Evidence from this review highlights the potential success of expanding the scope of practice for a range of health professionals and paraprofessionals, including pharmacists, dental therapists and midwives (47, 48, 51, 55, 56). However, further high-quality studies are required to examine these strategies on a broader scale. This scoping review also identified four studies using dental student staffed clinics which demonstrated effective and sustainable service delivery over several years (38, 39, 43, 44). This is consistent with data from other developed countries (UK), further supporting the scalability of student led dental clinics (73, 74). Managing OH among the elderly is a global challenge (75), with older Australians facing increased burden and barriers to accessing dental services (21, 76). Here too, expanding the scope of practice for nurses and oral health therapists may offer a potential solution. As a quantitative descriptive study in this review (2017) highlighted the successful scaling of a similar program expanding the scope of practice of dental therapists across ten residential age care facilities, engaging with 607 older adults (62). Additionally, this review showed some benefits with combining technologically enabled care and expanded scope of practice for oral health therapists to address service access gap in underserved areas (77). These approaches might be further enhanced by employing a range of dental workforce groups, such as oral health therapists, dental hygienists, dental therapists and other appropriate health workforce (public health midwives, prosthetists, dental assistants, community pharmacists) to optimise care access. Moreover, maintaining proper OH is very important for managing chronic diseases such as diabetes, heart disease and stroke among adults as these conditions are closely linked to maintaining their overall health and well-being (78). However, only three studies in this review described providing publicly funded care for eligible people with chronic diseases (57, 58, 65). Notably, these interventions were not sustained over time.

This review, while highlighting some successful approaches to improving access to oral healthcare through modifications of workforce or related service delivery outcomes, also highlighted a relative gap of scalable, long-term approaches to addressing these challenges. To address these workforce challenges, it is crucial to establish culturally accepted and scalable but contextually adaptable strategies to ensure sustainability. Workforce development, as emphasised in the National Oral Health Plan may be important for meeting future public OH goals (21). While more broadly effective population-level interventions designed by national and local policymakers are needed to meet the OH needs of cultural groups, including First Nations Australians (21), they fall beyond the scope of this review. However, addressing barriers at individual, organisational and system levels could contribute to a more equitable oral health landscape. Despite its relative wealth and high-quality health services, Australia demonstrates persistent inequalities within oral healthcare that have not been systematically addressed (79). This review highlights the need for greater capacity and infrastructure support to conduct large scale randomised controlled trials and related approaches to assess and develop high quality evidence that could potentially address this problem.
