# Supplementary material for: Exploring strategies for enhancing access to oral healthcare for adults in Australia: a scoping review
Source: Front Oral Health. 2025 Nov 6;6:1669597. doi: 10.3389/froh.2025.1669597 (PMC12631638; doi:10.3389/froh.2025.1669597)
Supplement: Supplementary file 1 [file Datasheet1.docx]

Supplementary information: Key characteristics of included sources

| **First author, year, citation number of reference** | **Title** | **Aims of the study/Objective of the work** | **Study setting** | **Study (project) participants** | **Study design and methods** | **Target population** | **Data collection tools** | **Interventions implemented/ proposed by the project** | **Effectiveness** |
| --- | --- | --- | --- | --- | --- | --- | --- | --- | --- |
| Calache et al., 2012 (51) | Provision of oral health care to adult patients by dental therapists without the prescription of a dentist | Evaluate a program designed to allow dental therapists with a university education to provide dental care within their existing scope of clinical practice to adult patients aged 26+ years, without the prescription from or supervision of a dentist | Public sector dental clinics in Victoria, Australia | University educated dental therapists with more than two years of service experience [total n=10] | Pre-post pilot study, using quantitative methods | Eligible patients of public sector aged 26 years and older who require dental treatment and are seen by dental therapists | Questionnaires including patient questionnaires, feedback forms for supervising and supporting dentists and assessments by supervising dentists during the clinical practicum | Educational bridging program for university educated dental therapists in Victoria, Australia. The educational program consisted of 42 hours of didactic content with lectures provided by clinical specialists and university academics, 14 hours of clinical observation, 42 hours of clinical practicum and 105 hours of clinical experience working in a community health centre. This educational program ran over a six-month period. | Post program feedback indicated that the university educated dental therapists effectively gained skills to treat adults independently |
| Christian et al., 2015 (49) | A paradigm shift in models of oral healthcare: An example and a call to action | Discuss the need for a paradigm shift in models of oral healthcare towards prevention-based, holistic, and evidence-informed approaches to address the social determinants of health and reduce the prevalence of oral diseases | Not for profit community health service in Victoria, Australia | No primary research participants | Evaluation study using qualitative methods | Aboriginal and Torres Strait Islander peoples, children (up to 17 years) and young people, homeless people and people at risk of homelessness, pregnant women who are healthcare or pension card holders, refugees and asylum seekers, registered clients of mental health and disability services | N/A | North Richmond Community Health-Oral Health (NRCH-OH) model of oral healthcare: An innovative preventive-based model of oral healthcare practice and services including health promotion, disease prevention, risk-based access to care, client- and family centred care, team-based provision of care, multidisciplinary care in three sites and two mobile outreach programs. This program provides services for public dental patients for subsidised fee and for private services for a fee. | Not reported |
| Conquest et al., 2017 (61) | Dental care for the elderly through a capped-fee funding model: Optimising outcomes for primary government dental services | Compare a capped payment formula for adults, to the fee-for-service model and the NSW Government services payment model; identify the presenting oral health needs of a 65+ years of age cohort during the period January 2011 to March 2015 | Public and private sector in Greater Southern health area, New South Wales, Australia | No primary research participants (de-identified patient records) | Descriptive, retrospective study using quantitative methods | Elderly patients aged 65 years and above | Deidentified elderly patient records from 2011 to 2015 (20 case studies) | Capped payment system named capped fee funding model: A state funded model used a voucher for elderly (65 years and above) patients to access timely dental care through the private sector. | Overall service model was ineffective, being more costly for older age groups |
| Durey et al., 2022 (63) | Community-centred oral healthcare for adults experiencing homelessness in Australia: Perceptions and experiences of key stakeholders | Examine whether providing free oral care using volunteer dental professionals and creating a responsive, respectful and trustworthy environment for clients who are homeless or at risk of homelessness is perceived to be effective in meeting their oral health needs | Community centred oral health clinics by volunteer dental professional in Fremantle, Western Australia, Australia | Purposively selected volunteer dental and other health professionals (n=13), homeless adults (n=18) and service providers (n=8) [total n=39] | Participatory Action Research using qualitative methods | Clients of community centres who are homeless or at risk of homelessness | Individual semi-structured interviews | Volunteer led oral healthcare model (St Patrick community support centre**)**: A community-based oral healthcare service provided by volunteers to adults experiencing homelessness. This includes flexible appointments, relatively short waiting time, text reminders/phone calls, stand by appointments following cancellations. | Interviews highlighted positive responses to the free clinic, especially quality of care and a safe and respectful environment |
| Fallon et al., 2006 (40) | Implementation of oral health recommendations into two residential aged care facilities in a regional Australian city | Introduce published evidence-based oral hygiene practices for patients with dementia in two publicly funded residential aged care facilities and monitor for changes in nurses awareness, knowledge, documentation and practice to improve patient outcomes and ensure appropriate accreditation standards were met; identify barriers to implementation of evidence-based recommendations and strategies to overcome these barriers | Publicly funded residential aged care facilities (two) in regional Queensland, Toowoomba, Australia | Patients, project stakeholders (senior dental therapist, oral health therapist, nurse educator and experts in quality improvement), day-to-day leaders (clinical nurse consultant and staff)  [total n=90] | Pre-post interventional pilot study using mixed methods | Residents of selected two public RACFs | Pre-post educational questionnaires (n=50) and oral audit tool (36), post implementation feedback sessions and follow up by semi structured focus groups | Implementing evidence-based oral hygiene practices for elderly patients with dementia: A quality improvement approach using PDCA cycle for residents with dementia in two publicly funded RACFs and monitor for changes in nursing awareness, knowledge, documentation and practice to improve patient outcomes and ensure appropriate accreditation standards. | This approach improved staff knowledge, care practices and care plans, with reported gains in resident’s oral health and use of best practice tools |
| Fung et al., 2024 (46) | A pilot study to assess the feasibility of real-time tele dentistry  in residential aged care facilities | Assess the feasibility of real-time tele dentistry conducted by aged care staff under live guidance from a dentist, through a narrative review of participant and clinician feedback; estimate the costs associated with different modes of tele dentistry compared to traditional on-site clinical examinations | Residential aged care facilities (two) in metropolitan Brisbane, Queensland, Australia | Residence in RACFs (n=16), Staff in RACFs (n=8)  [total n=24] | Pre-post comparative study using mixed methods | Residents in public RACFs | Questionnaire, in-depth interviews (n=24), chart audit tool and field notes | Implementation of real-time tele dentistry assessments conducted by RACF staff using intraoral cameras under live, remote guidance from a dentist | This intervention demonstrated both feasibility and cost-effectiveness of tele dentistry compared to traditional face-to-face dental examinations for aged care residents |
| Gardiner et al., 2020 (59) | Rural and remote dental care: Patient characteristics and health care provision | Determine the RFDS patient demographics and dental procedures conducted within rural and remote Australia; determine the dental service provision and coverage (RFDS and non-RFDS) within mainland rural and remote Australia | RFDS dental clinics located in rural and remote settings, Australia | No primary research participants | Retrospective cohort study using quantitative methods | Rural and remote communities without services of any other dental programs | Past health administrative data of patients who accessed a RFDS dental clinic from April 2017 to September 2018 | RFDS mobile dental service in rural and regional settings in Australia: The services provided using fly-in fly-out, mobile and outreach delivery models funded by the Commonwealth of Australia. | Intervention increased service uptake and improved access for rural populations despite travel barriers |
| George et al., 2016 (47) | The evaluation of an oral health education program for midwives in Australia | Evaluate the effectiveness of an online Midwifery Initiated (MIOH) Oral Health education program in improving the oral health knowledge and confidence of midwives in promoting maternal oral health | Public sector hospitals in New South Wales and Victoria, Australia | Midwives: New South Wales (n=17) and Victoria (n=33)  [total n=50] | Pre-post pilot study using quantitative methods | Eligible pregnant women for public oral health services | Pre-post questionnaires | MIOH education program: An online evidence-based education program systematically developed for midwives as a professional development activity. The program contained three self-paced modules focussing on various aspects of perinatal oral health including the oral health screening and referral process as well as a theoretical and practical skill assessment. | The program significantly improved midwives' oral health knowledge, with most feeling confident in promoting oral health and referring women to dental services |
| Heilbrunn-Lang et al., 2015 (48) | Midwives' perspectives of their ability to promote the oral health of pregnant women in Victoria, Australia | Explore the perspectives of midwives in Victoria towards incorporating oral health promotion into their antenatal practice after undergoing training through the MIOH online education program | Public hospitals and Aboriginal Community Controlled Health Services Victoria, Australia | Midwives  [total n=39] | Pre-post pilot evaluation using mixed methods | Eligible pregnant women for public oral health services | Pre-post-questionnaire containing open ended items and feedback forms | MIOH education program: An online evidence-based education program systematically developed for midwives as a professional development activity. The program contained three self-paced modules focussing on various aspects of perinatal oral health including the oral health screening and referral process as well as a theoretical and practical skill assessment. | The program increased midwives' confidence in promoting oral health with most finding it valuable and feasible for antenatal care |
| Ingram et al., 2024 (56) | Evaluation of the empower model of care (EMC) for partial denture clients in a public oral health care setting | Evaluate the effectiveness of a community-based oral health program to improve a patient’s long-term oral health behaviours assessed by evaluating if patients return to have additional teeth added to their removable partial dentures (RPD) after completing the EMC | Monash Dental Services (MHDS) in Melbourne, Australia | Public oral health clients receiving removable partial dentures. Pre intervention group (n=2034), intervention group (n=584)  [total n=2618] | Descriptive, retrospective study using quantitative methods | Eligible adult patients of public sector for partial removable dentures | Titanium electronic database records (n=2618), client admission forms, clinical oral health assessments, and item code tracking for denture additions | Empower model of care, an evidence-based, multifaceted approach, which is prevention and outcome-focused leading to a well-informed, empowered clients | This new model of care was effective about the clinical evidence of the effectiveness of the Empower model and methodology in improving clinical outcomes |
| Kelly et al., 2024 (65) | Codesigning culturally safe oral health care with First Nations kidney warriors experiencing kidney disease in South Australia | Codesign strategies to address disparities and gaps in care and co-create more accessible, responsive, culturally safe and sustainable models of care together | Public health sector of metropolitan Adelaide, South Australia, Australia | Purposively selected First Nations kidney warriors (n=10), dental hygienist students (n=17), oral health and kidney health professionals, educators, and service providers (n=12)  [total n=39] | Participatory Action Research (pre-post pilot study) using qualitative methods | First Nations people (Aboriginal and Torres Strait Islander individuals) living with kidney disease | Pre-post placement surveys, clinical yarning, community consultations, collaborative discussions and reflections, informal feedback and observations | Culturally safe oral health education and clinical placement model: A Co-designed student placements at Kanggawodli Hostel, including cultural safety training, clinical yarning and two-way knowledge sharing | The intervention was effective in enhancing cultural safety, oral health literacy and access to care |
| Kong et al., 2021 (60) | Aboriginal health workers promoting oral health among Aboriginal and Torres Strait Islander women during pregnancy: Development and pilot testing of the Grinnin’ Up Mums and Bubs program | Develop an evidence-based, culturally appropriate oral health model of care for pregnant women; pilot test the model of care with Aboriginal health workers to identify the acceptability and satisfaction of the model of care, any improvements in their oral health knowledge and confidence and future recommendations | Public sector of Greater Western Sydney in New South Wales, Australia | Aboriginal health workers [total n=7] | Participatory Action Research (pre-post pilot study) using mixed methods | Aboriginal pregnant women eligible for public dental services, ACCHSs or private sector | Focus groups, interviews and pre–post pilot questionnaire | The Grinnin’ Up Mums & Bubs Model of Care: A culturally safe program designed for pregnant women. The program comprises three phases, preparation, knowledge making and giving (piloting). Referrals of indicated pregnant women to oral health practitioners (public or private) were one of the main components of this intervention. | Participants were satisfied with the model, showing improved knowledge, confidence and recommending better oral health access |
| Kruger et al., 2010 (64) | Sustaining oral health services in remote and Indigenous communities: A review of ten years experience in Western Australia | Highlight the disparities in access to dental care faced by rural, remote, and First Nations populations in Western Australia; discuss the sustainable model developed by the Centre for Rural and Remote Oral Health to address these challenges through integrated education, service and research | Rural and remote areas of Western Australia, Australia | No primary research participants | Descriptive, retrospective study using Qualitative methods | Rural, remote and First Nations communities who face challenges in accessing oral health services | Past health data (ten years) on different service, research and education initiatives | Sustainable model developed by the Centre for Rural and Remote Oral Health (CRROH) through a vertically integrated service, education and research driven model: Sustaining oral health services in remote and First Nations communities with an emphasis on the oral health needs of First Nations Australians. | Not reported |
| Lam et al., 2015 (58) | Conundrums in merging public policy into private dentistry: Experiences from Australia's recent past | Highlight issues surrounding access and affordability to dental care, the impact of inadequate dental care on the public health system, public policies implemented to improve access, and the unintended consequences of policy amendments considering CDDS and Medicare teen dental plan | Nationwide oral health program, Australia | No primary research participants (publicly available 2007-2009 CDDS data) | Descriptive, retrospective study using quantitative methods | People with chronic medical conditions (diabetes, cardiovascular disease, autoimmune disorders or other chronic illnesses) that impacted oral health | N/A | CDDS in Australia: A government subsidy program for private dental treatment for individuals suffering from chronic illnesses impacting their oral health through Medicare funding. The referrals of the indicated patients by medical practitioners to private dental clinics were required. | This was ineffective due to poor gatekeeping mechanisms, leading to increased referrals, cost blowouts, ultimately resulting in program termination |
| Lin et al., 2022 (53) | A tele dentistry pilot study on patient initiated care | Describe DHSVs patient initiated tele dentistry model of care implemented during peak COVID transmission in Victoria | Public dental health services in Victoria, Australia | 2493 patients | Descriptive, retrospective pilot study using quantitative methods | Vulnerable populations (include low-income communities, Aboriginal and Torres Strait Islander people, people who are homeless or at risk of homelessness, pregnant women, refugees and asylum seekers, and people registered with mental health and disability services) | Patient records of telehealth appointment (between May 1, 2020 and April 30, 2021, 2492 patients) | Dental Health Services Victoria’s (DHSV) patient initiated tele dentistry to provide remote oral healthcare services including referrals for indicated patients during the COVID-19 pandemic in public setting. | This was effective, with 87 to 90% of patients reporting satisfaction, feeling their needs were met, questions answered and being involved in their treatment |
| Mangoyana et al., 2023 (44) | Positive oral health outcomes: A partnership model improves care in a rural Indigenous community | Explore the benefits of a partnership between a university dental school and a Community Controlled Health Service, specifically in relation to improving the oral health of an underserved rural First Nations community | Community controlled health services in Dalby (rural town), South-West Queensland, Australia | Purposively selected local community elders, GHS community health support group members and GHS management staff and staff for five focus groups  [total n=38] | Descriptive study using qualitative methods | First Nations communities eligible for public oral health services | Semi structured focus group sessions (five) | Establishment and operation of dental student outplacement clinics (student led clinics) in regional Queensland: The University of Queensland School of Dentistry partnered with GHS, a federally funded Aboriginal and Torres Strait Islander Community Controlled Health Service (ATSICCHS) to offer free dental care to First Nations communities. | This partnership effectively improved oral health awareness and access for the First Nations community, benefiting both community and dental students |
| March et al., 2023 (43) | Positive impacts of oral health services provision by a student led primary care clinic to an Australian rural Indigenous community | Explore impacts of the Dalby dental student outplacement clinic on population oral healthcare provision and access | Community controlled health services in Dalby (rural town), South-West Queensland, Australia | No primary research participants (aggregated and de-identified 2017, 2018 and 2019 student led clinic services data) | Descriptive, retrospective study using quantitative methods | Rural First Nations communities eligible for public oral health services | N/A | Establishment and operation of dental student outplacement primary care clinics (student led) in regional Queensland targeting Goondir Health Services (GHS) and ATSICCHS: Partnership between University of Queensland and GHS to reduce the pressure on the public system. Free dental care is provided at the Dalby student led clinic that operates with support from the University of Queensland School of Dentistry. | Results from 4536 services over three years showed a shift from acute care to preventative care, indicating improved community oral health. Government public waiting list times decreased, reflecting reduced pressure on the system |
| McPhee et al., 2021 (54) | Understanding the barriers and enablers to Minimal Intervention Dentistry in an Australian community dental agency | Explore staff perspectives on the barriers and enablers impacting the implementation of minimally invasive dentistry (MID) practice in an Australian public dental program | Public community dental services (two) in Victoria, Australia | Dentists (n=4), dental/oral health therapists (n=6), dental assistants (n=10) and  oral health coaches (n=4)  [total n=24] | Descriptive study using qualitative methods | Public oral service consumers | Semi structured interviews | MID in public community dental program: An approach to dental care that aims to preserve as much tooth structure as possible, using preventive and minimally invasive techniques to manage dental caries and other oral health conditions. | Not reported |
| Meldrum et al., 2018 (52) | The role of community mental health services in supporting oral health outcomes among consumers | Explore the role of Australian community mental health services in supporting the prevention and management of poor oral health among people living with mental illness | Community mental health service (Neami National) with partnership of dental school, University of Melbourne Victoria, Australia | Individuals with severe mental illness receiving services from community mental health programs (females n=8 and males n=4)  [total n=12] | Descriptive explanatory study using qualitative methods | Individuals with severe mental illness | Focus groups (two) (n=8), semi-structured interviews (n=4) | Health Prompt Tool: A health prompt in the form of a physical health questionnaire designed. Use of this tool, which included a question about dental check-ups, to initiate conversations about oral health and support engagement with dental services among individuals living with mental illness as a priority access. | The intervention effectively improved oral health support, education and access for individuals with mental illness |
| Oldroyd et al., 2017 (50) | Program evaluation of the inner south community health oral health program for priority populations | Evaluate the impact of an oral health program on disadvantaged populations, focusing on trust-building, challenges in implementation and the need for policy changes to improve access to care | Inner South community health services of Melbourne, Victoria, Australia | Client consumers (n=29) for questionnaire, eight in-depth interviews (n=8) and four focus group discussions (m=31) with either clients, healthcare worker or partner agencies  [total n=68] | Descriptive, retrospective evaluation study using mixed methods | Priority population (people experiencing homelessness, people living with HIV or mental illness, those living in pension, people with alcohol and other drug problems and First Nations Australians) exempt from the usual waiting lists | Satisfaction questionnaire (29 clients),  focus groups (four) in depth interviews (eight) | Oral Health Program for Priority Populations (OHPPP): A public dental service, that focused on building trust and providing inclusive oral healthcare for disadvantaged individuals. The OHPPP provides eight clinics for preventative and restorative dental care across two clinical sites. | The OHPPP was effective, with 92% of clients reporting improved health and strong support for its value and accessibility despite implementation challenges |
| Stelfox et al., 2025 (55) | Evaluating and expanding Rural Victorian community pharmacists role in providing oral healthcare | Investigate rural pharmacists' perceptions of oral health services they provide in towns without dental practitioners and how to best expand their role | Rural towns in central Victoria, Australia | Community pharmacists (n=11) | Descriptive study using qualitative methods | Regional residents with no access to local dental practitioners | Semi-structured telephone interviews | Expanding rural pharmacists' roles to include oral health advice, consultations and collaboration with dental practitioners, supported by training and promotional resources | Intervention demonstrated that rural pharmacists can effectively support oral health promotion, improving access and awareness in communities lacking dental practitioners |
| Stormon et al., 2018 (38) | Does a facilitated pathway improve access to dental services for homeless and disadvantaged adults? | Evaluate an innovative system integration model for clients of homeless services in Brisbane, Australia | Community organisations (ten) in Brisbane, Queensland, Australia | Clients experiencing homelessness in community organisations  [total n=76] | Interventional pilot study with multi-method approach using mixed methods | Adult homeless Clients of community centres | Questionnaires (including open ended questions) and oral health screenings, and follow-up feedback surveys | System integration model for oral healthcare: A collaborative project between the University of Queensland School of Dentistry and Metro North HHS for the provision of onsite screenings and referral for dental treatment appointment at a public dental facility within the same week. | This intervention was effective, with 85% of participants attending dental appointments and reporting positive experiences |
| Stormon et al., 2021 (39) | Facilitating access to dental care for people experiencing homelessness | Evaluate three models aimed at improving access to dental care for people experiencing homelessness by assessing participant characteristics, model effectiveness, resource use and value for money | Community organisations (four) in Brisbane, Queensland, Australia | Clients experiencing homelessness in four community organisations: Model 1(n=76), model 2 (n=66) and model 3 (n=43)  [total n=185] | Descriptive pilot study using quantitative methods | Adult homeless Clients of community centres | Questionnaires, costs assessment from both a community organisation and health services perspective, as well as estimations based on resource use measured during model trials | Facilitated three access models developed and implemented at selected community organisations: A collaborative project between the University of Queensland School of Dentistry and Metro North HHS for homeless adult communities. | Model 1 was the most effective (84.2% attendance), while Model 3 was the most cost effective, increasing access to dental care for people experiencing homelessness |
| Stormon et al., 2022 (45) | SMS reminders to improve outpatient attendance for public dental services: A retrospective study | Build on existing literature and determine whether implementing an SMS reminder system for dental appointments in a low SES area increases attendance rates and decreases unable to attend (UTA) and failure to Attend (FTA) rates | West Moreton Hospital and Health Services in Ipswich, Queensland, Australia | No primary research participants (Consumers receiving services at public dental clinics before and after implementation of SMS reminders, 10,000 records) | Retrospective pre-post interventional study using quantitative methods | Public oral service consumers | Pre-post institutional data (10,000 Patient appointment records before and after implementation of SMS reminders) | Sending SMS reminders to public oral health attendants one business day prior to scheduled appointment, in addition to the appointment letter. Patients are asked to confirm their attendance by replying to the SMS for confirming for the appointment. | This intervention was ineffective overall with only slight improvements in attendance and increased FTA rates across services |
| Tynan et al., 2018a (41) | Integrated approach to oral health in aged care facilities using oral health practitioners and tele dentistry in rural Queensland | Improve access to oral health services for residents in rural and regional aged care facilities through an integrated model of care using oral health therapists and tele dentistry | Residential aged care facilities (four) in Darling Downs Hospital and Health Services, Queensland, Australia | Residence in RACFs  [total n=116] | Pre-post evaluation study using quantitative methods | Residents in public RACFs | Chart audits tool (pre and post chart audits), pre-post consultations with key stakeholders and field nots | Implementation of integrated model of care for through quality improvement imposing oral health therapists (for screening) and tele dentistry in regional (one) and rural (three) RACFs. | This intervention was effective, improving care plan implementation, reducing dental visits and showing potential cost savings |
| Tynan et al., 2018b (42) | An integrated oral health program for rural residential aged care facilities: A mixed methods comparative study | Investigate the impact and experience of this integrated approach to oral health compared to current standard care within regional and rural RACFs | Residential aged care facilities (six) and Multi-Purpose Health Services (three) in regional Queensland, Australia | Residence in RACFs (n=300) audits, key staff informants and managers for in depth interviews and focus groups  [total n=300] | Pre-post comparative study using mixed methods | Residents in public RACFs | Focus group (one) with key staff and managers, in-depth Interviews (eight) with key staff and managers, questionnaire, chart audits tool (252 audits) and field notes | Implementation of integrated oral health program (oral health therapist and tele dentistry centred) for rural RACFs through quality improvement: A multidisciplinary approach incorporating oral health professionals and RACF staff for improving access. | This integrated program was effective, improving compliance with care standards and supporting better oral health management for residents |
| Weerakoon et al., 2014 (57) | An Australian government dental scheme: Doctor-dentist-patient tensions in the triangle | Explore the experiences and tensions between patients, doctors, and dental practitioners within the Australian Chronic Disease Dental Scheme | Nationwide oral health program, Australia | Dentists, doctors and CDDS patients [total n=31] | Descriptive study using qualitative methods | People with chronic medical conditions (diabetes, cardiovascular disease, autoimmune disorders or other chronic illnesses) that impact oral health | Focus groups (two) and in-depth semi structured interviews (24) | CDDS in Australia: A government subsidy program for private dental treatment for individuals suffering from chronic illnesses impacting their oral health through Medicare funding. The referrals of the indicated patients by medical practitioners to private dental clinics were required. | This was ineffective due to poor gatekeeping mechanisms, leading to increased referrals, cost blowouts, ultimately resulting in program termination |
| Wright et al., 2017 (62) | Residential age care and domiciliary oral health services: Reach-OHT, the development of a metropolitan oral health programme in Sydney, Australia | Develop an on-site oral health assessment, education and service delivery programme to improve access to oral healthcare for older people living in RACFs | Residential aged care facilities (ten) in Sydney, New South Wales, Australia | Residence in RACFs [total n=607] | Descriptive observational study using quantitative methods | Residents in public RACFs who were full or part pension cardholders | Oral Health Assessment Tool (OHAT) | Reach-OHT program, major coordinating and monitoring role of oral health therapists in the programme developed to provide oral healthcare to older people in RACFs: A comprehensive oral healthcare programme implemented in RACFs built around six core elements. | This was effective with 75% of residents receiving assessments and 46.5% receiving dental care, improving oral health and staff education |
| Australian Dental Health Foundation, 2020 (36) | Australian dental health foundation, pre-budget submission 2020-2021 | Improve oral health outcomes for disadvantaged and underserved Australians by providing access to dental care, supporting dental professionals and promoting oral health education | Rural, remote and regional settings in Australia | No primary research participants | Mixed methods | Disadvantaged population (individuals facing financial, geographic, or social barriers including those affected by domestic violence, substance abuse, homelessness, or low socioeconomic status), Aboriginal and Torres Strait Islander communities, high-risk communities (populations in high-risk or underserved areas) | N/A | Pro-bono dental services: Dentists volunteer their time and skills to provide free dental care to clients referred by registered charities or non-profit organisations, targeting those in domestic violence, substance abuse recovery, homelessness or low socioeconomic conditions.  Indigenous scholarship program: Aboriginal and Torres Strait Islander students in dental hygiene or oral health therapy, aiming to encourage them to serve their communities.  Community service grants: Partner with Mars Wrigley corporation to fund community-based oral health projects and support volunteer dentists and dental students in high-risk areas. | Not reported |
| Battye et al., 2022 (37) | Increasing dental and oral health training in rural and remote Australia: Feasibility study: Final report | Determine the feasibility and best approach to increasing dental and health training through the RHMT program into more rural and remote locations; consider the best benefits to service delivery to local communities and inform future program design and government policy to support Australia’s future rural health workforce | Rural, remote and regional settings in Australia | No primary research participants | Mixed methods | Rural, remote, and regional communities, dental and oral health students who will be trained and placed in rural settings, Aboriginal and Torres Strait Islander students, early career professionals (new graduates transitioning into rural dental and oral health roles) | N/A | National summit to focus on rural and remote dental and oral health workforce and training.  Evidence-based training: Align dental and oral health training requirements with evidence specific to rural practice.  Early career support: A program to support rural graduate transition into early career roles.  Enhancing supervision capacity of graduates for rural placements: Building academic capacity to support rural and remote training.  Embed oral health training within University Departments of Rural Health (UDRHs).  Clinical school: Establishing rural dental and oral health clinical schools.  First Nations workforce: Promote and grow the Aboriginal and Torres Strait Islander dental and oral health workforce. | Not reported |

ACCHSs: Aboriginal Community Control Health Services, CDDS: Chronic Disease Dental Service, HHS: Hospital and Health Services, PDCA: Plan Do Check Act, RACF: Residential Aged Care Facility, Reach-OHT: Reach Oral Health Therapist, RFDS: Royal Flying Doctors Service, RHMT: Rural Health Multidisciplinary Training
